# Spatial distributions at equilibrium under heterogeneous transient subdiffusion

**DOI:** 10.3389/fphys.2014.00437

**Published:** 2014-11-12

**Authors:** Hugues Berry, Hédi A. Soula

**Affiliations:** ^1^Beagle, Institut National de Recherche en Informatique et en AutomatiqueVilleurbanne, France; ^2^Laboratoire d'InfoRmatique en Image et Systèmes d'information, UMR 5205 CNRS-INSA, Université de LyonVilleurbanne, France; ^3^Institut Multidisciplinaire de Biochimie des Lipides, Inserm UMR1060, Université de LyonVilleurbanne, France

**Keywords:** brownian diffusion, subdiffusion, spatial protein distribution, nonhomogeneous cellular media, continuous-time random walks

## Abstract

Experimental measurements of the mobility of macromolecules, especially proteins, in cells and their membranes consistently report transient subdiffusion with possibly position-dependent—non-homogeneous—properties. However, the spatiotemporal dynamics of protein mobility when transient subdiffusion is restricted to a subregion of space is still unclear. Here, we investigated the spatial distribution at equilibrium of proteins undergoing transient subdiffusion due to continuous-time random walks (CTRW) in a restricted subregion of a two-dimensional space. Our Monte-Carlo simulations suggest that this process leads to a non-homogeneous spatial distribution of the proteins at equilibrium, where proteins increasingly accumulate in the CTRW subregion as its anomalous properties are increasingly marked. In the case of transient CTRW, we show that this accumulation is dictated by the asymptotic Brownian regime and not by the initial anomalous transient dynamics. Moreover, our results also show that this dominance of the asymptotic Brownian regime cannot be simply generalized to other scenarios of transient subdiffusion. In particular, non-homogeneous transient subdiffusion due to hindrance by randomly-located immobile obstacles does not lead to such a strong local accumulation. These results suggest that, even though they exhibit the same time-dependence of the mean-squared displacement, the different scenarios proposed to account for subdiffusion in the cell lead to different protein distribution in space, even at equilibrium and without coupling with reaction.

## 1. Introduction

Traditional biology and biochemistry approaches tend to view the inside of a cell and its constituent membranes as uniform, homogeneous and well-stirred media. However, under the light of the recent advances in experimental methodologies, they rather appear disordered and heterogeneous, with high levels of crowding typical of poorly-connected media (Dix and Verkman, 2008; Cambi and Lidke, 2012; Höfling and Franosch, 2013; Parry et al., 2014). For instance, cell membranes are heterogeneous collections of contiguous spatial micro- or nanodomains with various length and time scales (e.g., fences, lipid rafts, caveolae) (Jacobson et al., 2007; Cambi and Lidke, 2012), that restrict the lateral mobility of proteins in a position-dependent way (Kenworthy et al., 2004; Goodwin et al., 2005; Fujita et al., 2007; Day and Kenworthy, 2009; Kusumi et al., 2011).

In addition to the complexity brought about by the spatial heterogeneity of protein mobility, protein diffusion itself can deviate from the ideal case of Brownian motion. Measurements of the movement of proteins in living cells (in particular in membranes) has consistently been reported to exhibit subdiffusion (a variety of anomalous diffusion). In subdiffusion, the mean square displacement scales sub-linearly with time, 〈*R*^2^(*t*)〉 ∝ *t*^γ^ with γ < 1 (Schwille et al., 1999; Smith et al., 1999; Fujiwara et al., 2002; Weigel et al., 2011; Höfling and Franosch, 2013), as opposed to γ = 1 in Brownian motion. Currently, there exist three major theoretical scenarios to explain the observations of subdiffusion, all of which rest on the idea that the interior of cells and their membranes experience large molecular crowding due to their high densities of proteins, lipids, carbohydrates, filamentous networks and organelles, with widely-distributed sizes (Dix and Verkman, 2008; Höfling and Franosch, 2013). The arguably simplest scenario, referred to as “Fractional Brownian Motion,” is a generalization of the classical Brownian motion, where the random increments between two successive locations of the random walker are not independent (like in Brownian motion) but present long-range temporal correlations (Barkai et al., 2012). The second scenario is hindered diffusion in the presence of randomly-distributed immobile obstacles (Saxton, 1994; Berry, 2002; Höfling and Franosch, 2013). The third scenario, referred to as “Continuous-Time Random Walks” (CTRW) assumes that the complexity of the cellular media changes the statistics of the residence time τ between two moves of the random walkers. Whereas Dirac—or exponentially—distributed residence times lead to the classical Brownian motion, power-law distributed residence time, η(τ)∝ τ^−α^, generates subdiffusive motion with γ = α − 1 provided 1 < α < 2 (Bouchaud and Georges, 1990; Metzler and Klafter, 2000; Höfling and Franosch, 2013). Those three scenarios all lead to sublinear scaling of the mean square displacement with time, i.e., subdiffusion. Other scenarios for subdiffusion have been explored (e.g., scaled Brownian motion, some heterogeneous Brownian processes or correlated CTRW) but are less well studied, see Metzler et al. (2014) for a review.

Whatever the underlying scenario considered, subdiffusion is usually studied in situations that are so simple that their applicability to biology can be questioned. Yet, several factors contribute to the complexity of the cellular media:

(*i*) *Subordination*: The three scenarios above need not be mutually exclusive but could combine in a subordinated process (Weigel et al., 2011; Tabei et al., 2013).

(*ii*) *Transience*: In several experimental reports (Platani et al., 2002; Murase et al., 2004; Saxton, 2007; Bronstein et al., 2009; Jeon et al., 2011), the anomalous regime is only transient: after the initial anomalous regime, the mean square displacement crossovers back to normal (Brownian) diffusion (with γ = 1) but with a smaller apparent diffusion coefficient.

(*iii*) *Non-homogeneity*: The intensity of the molecular crowding, and/or the anomalous exponent γ may vary depending on the location inside the cell (Wachsmuth et al., 2000; Kühn et al., 2011), thus defining a position-dependent exponent γ(*x*).

The spatiotemporal dynamics of protein mobility when any of those three factors is at play is still obscure. For instance, it is only recently that subdiffusion with space-dependent exponent has been explored. In a one-dimensional lattice-based space where the anomalous exponent is set to a much smaller value in one of the lattice sites, Fedotov and Falconer (2012) reported a striking accumulation phenomenon: after possibly a long transient, all the mobile molecules locate at the lattice site with smallest exponent. Similar accumulation phenomena were reported by Korabel and Barkai for particle transport in binary systems, for which space is partitioned into two subdomains, where diffusion is Brownian or CTRW, respectively (Korabel and Barkai, 2010, 2011). Similarly, recent studies have shown that non-homogeneous Brownian motion (where the diffusion coefficient depends on space) can give rise to counterintuitive behaviors, including CTRW-like transport (Cherstvy et al., 2013, 2014).

**Figure 1:**
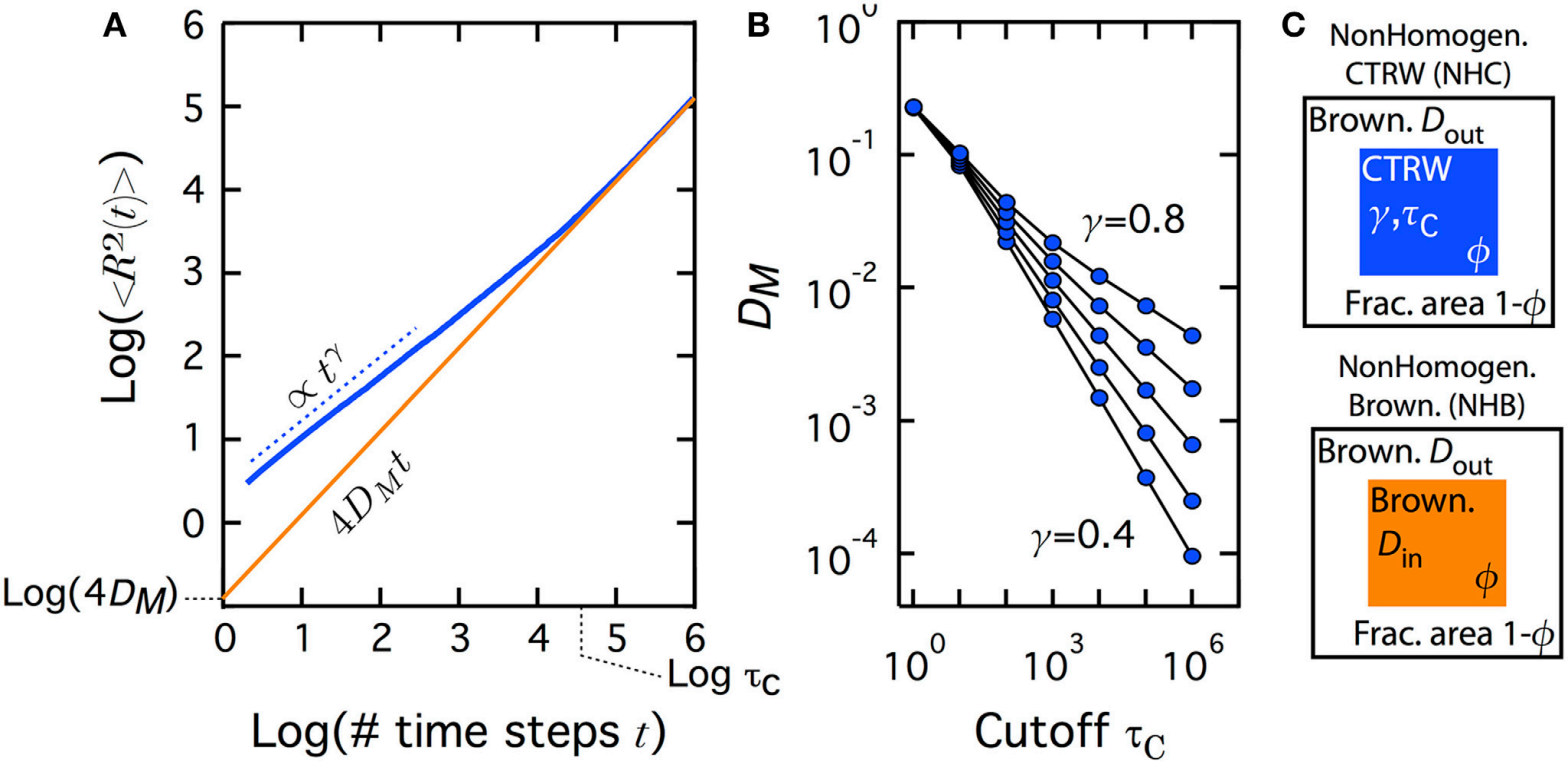
**Transient subdiffusion (CTRW) turns Brownian at times longer than the cutoff τ_*C*_**. **(A)** The mean square displacement 〈*R*^2^〉 in transient CTRW (*blue* curve) first scales as *t*^γ^ (where γ is the anomalous exponent), but at time scales larger than the cutoff τ_*C*_, transient CTRW converges to a Brownian motion with *D*_*M*_ as (macroscopic) diffusion coefficient, i.e., 〈*R*^2^〉 = 4*D_M_t* (*brown* curve). The dashed line shows a scaling with exponent γ, 〈*R*^2^〉 ∝ *t*^γ^. **(B)** The microscopic diffusion coefficient *D_M_* decreases rapidly with increasing cutoff times τ_*C*_ and decreasing anomalous exponents. γ = 0.8, 0.7,0.6,0.5, and 0.4, from top to bottom. **(C)** In the following, we study the spatially non-homogeneous case were the diffusion conditions inside a central patch, of fraction area ϕ differ from the diffusion conditions outside the patch. In the non-homogeneous CTRW (NHC) case, diffusion is Brownian with diffusion coefficient *D*_out_ outside the patch and a CTRW with parameters (γ, τ_*C*_) inside. In the non-homogeneous Brownian (NHB) case (used for comparison), diffusion is Brownian both outside the patch (diffusion coefficient *D*_out_) and inside the patch (with diffusion coefficient *D*_in_ set so as to match the macroscopic diffusion coefficient *D*_*M*_ obtained in transient CTRW with the same parameters γ and τ_*C*_).

In the present work, we focused on CTRW-based subdiffusion and studied the impact of transience and non-homogeneity on the spatial distribution of the proteins at equilibrium. In the framework of CTRW, transience is naturally introduced by the addition of an upper bound to the residence time τ_*C*_ (cutoff time, see Materials and Methods). In such a transient CTRW, 〈*R*^2^〉 indeed first scales as *t*^γ^ at short times, then crosses over to a Brownian motion (with γ = 1) for *t* ≫ τ_*C*_. This asymptotic Brownian regime can be considered a macroscopic view of the underlying microscopic subdiffusion, with a “macroscopic” diffusion coefficient (Figures 1A,B):

(1) $$D_{M}=\frac{{\left(\Delta x\right)}^{2}}{4\int_{\Delta t}^{\tau_{C}}\tau \eta (\tau )d\tau }$$

where η(τ) is the distribution of the residence time, Δ*t* the time step of the Monte-Carlo simulation and $\int_{\Delta t}^{\tau_{C}}\tau \eta (\tau )d\tau$ is the mean residence time of the random walk. To introduce non-homogeneous diffusion, we considered a “patchy” two dimensional space domain (Figure 1C). In the center of the domain, we locate a square patch, of area fraction ϕ, in which diffusion is due to a transient CTRW. Outside the patch, diffusion is Brownian. This setup therefore defines a non-homogeneous transient CTRW (NHC) process.

Our Monte-Carlo simulations show that the spatial distribution at equilibrium of proteins subject to such a transient NHC process is itself non-homogeneous: with increasingly strong subdiffusion (i.e., when γ decreases or τ_*C*_ increases), the proteins progressively accumulate in the central patch. We show that this accumulation is totally controlled by the long-time (Brownian) regime of the transient CTRW and not the initial anomalous transient. However, we also show that the dominance of the long-time Brownian regime in transient subdiffusion cannot be extended to other scenarios. Indeed, non-homogeneous transient subdiffusion due to hindrance by randomly-located immobile obstacles also exhibit transient subdiffusion followed by a slowed-down Brownian regime. However, we show that this situation does not lead to such a strong local accumulation, but to depletion of the central patch or weak accumulation, depending on how concentrations are computed. Therefore, even in the simplest case of pure lateral mobility (i.e., no reaction), the knowledge of the time-dependence of the mean square displacement is not sufficient to predict the distribution of the proteins at equilibrium. This simulation work strongly suggests that the different scenarios proposed to account for transient subdiffusion in the cell could lead to different protein distribution in space, even at equilibrium and in the absence of any reaction (binding, post-translational modifications, internalization…).

## 2. Materials and methods

All simulations take place in a *w* × *w* 2D square lattice with reflective boundaries. At initialization, we position *N_T_* proteins uniformly at random without excluded volume i.e., several proteins can occupy the same site. When a protein arrives at lattice site (*i*,*j*) at time *t*_arrival_, its residence time τ is sampled from a distribution η_*i*,*j*_. The next jump time of the protein therefore is set as *t*_arrival_ + τ. η_*i*,*j*_ depends on the nature of the arrival site (*i*,*j*). If (*i*,*j*) belongs to an area of Brownian motion, η_*i*,*j*_ is an exponential distribution of parameter τ_*B*_Δt (where Δ*t* is the simulation time step): η_*i*,*j*_(τ) = 1/(τ_*B*_Δ*t*) exp (− τ/(τ_*B*_Δ*t*)). τ_*B*_Δ*t* is the average residence time and sets the diffusion coefficient at site (*i*,*j*): *D*(*i*,*j*) = Δ*x*^2^/(4τ_*B*_Δ*t*) where Δ*x* is the lattice spacing (see Equation 1). If the site (*i*,*j*) belongs to an area of CTRW, the residence time is sampled from the power-law distribution η_*i*,*j*_(τ) = γτ^−(1+γ)^/(Δ*t*^ − γ^ − τ*_c_*^−γ^), for which $\int_{\Delta t}^{\tau_{c}}\eta (\tau )d\tau =1$. Hence Δ*t*, the simulation time step is the smallest residence time possible and τ*_c_*, the cut-off time, sets its maximal value. At each simulation time step *t* = *n*Δ*t*, the algorithm finds all the proteins that have their jump time in [*n* − 1, *n*]Δ*t*. Each of those proteins independently jump to a destination site that is chosen uniformly at random from its 4 nearest neighbors (*i* ± 1, *j* ± 1).

Note that in the literature, the cutoff of the residence time distribution is frequently introduced using a soft cutoff, instead of the hard cutoff used in this study. Such “tempered” (soft) cutoff is commonly obtained by adding an exponential cutoff to the distribution of residence times i.e., η(τ) = γΔ*t*^γ^ τ^−(1+γ)^ exp (− τ/τ_*C*_). In a subset of the simulations shown below, we have replaced our hard cutoff with a tempered one and found that the results were identical to those obtained with our hard cutoff. We conclude that the exact implementation of the cutoff does not have significant impact on the results reported below.

For simulation of spatially non-homogeneous Brownian diffusion (NHB), we position the boundary of the slowed-down patch in the middle of neighbor lattice sites. Each lattice side (*i*,*j*) therefore belongs either to the slowed-down patch [we thus set its diffusion constant to *D*(*i*,*j*) = *D*_1_] or to the outer region (and we set *D*(*i*,*j*) = *D*_0_ > *D*_1_). In the case of spatially non-homogeneous CTRW (NHC), we also position the boundary between the CTRW patch and the Brownian exterior in the middle of neighbor lattice sites. Therefore each lattice side (*i*,*j*) either belongs either to the CTRW patch or to the outer Brownian region.

To compare NHB and NHC, we simply computed the average residence time for the CTRW distribution according to the cutoff (τ*_c_*) and γ parameters: $\int_{\Delta t}^{\tau_{c}}\tau \eta (\tau )d\tau$ and use this value as the mean residence time for Brownian motion (see Equation 1).

Subdiffusion due to obstacle hinderance was simulated by positioning obstacles at random locations (with uniform distribution) within the inner region at the beginning of the simulation. In this instance, obstacles behave a separate type of molecules that are kept unreactive and immobile. Moreover, they exclude the lattice site they occupy: whenever the chosen destination site of a moving protein contains an obstacle, the protein is reflected back to its origin site (the destination site becomes the origin position).

Standard parameter values were used throughout the article, unless otherwise specified: lattice size *w* = 800, Δ*t* = 1, Δ*x* = 1 and diffusion coefficient *D*_out_ = 1/4. Every simulation was initiated with *N*_T_ = 10^4^ proteins and was run until the density of proteins reaches equilibrium. Depending on simulation conditions, equilibrium was typically reached after at most 10^5^ (obstacles) to 5 × 10^5^ (slowed down Brownian diffusion or CTRW) time steps. The number of proteins present in the patch at equilibrium, *N*_in_, was therefore computed as a time-average for *t* ∈ [9.5, 10.0] × 10^5^. Each simulation was repeated 20 times with different realizations of the pseudo-random numbers. The data presented below are averaged over those 20 runs.

## 3. Results

**Figure 2:**
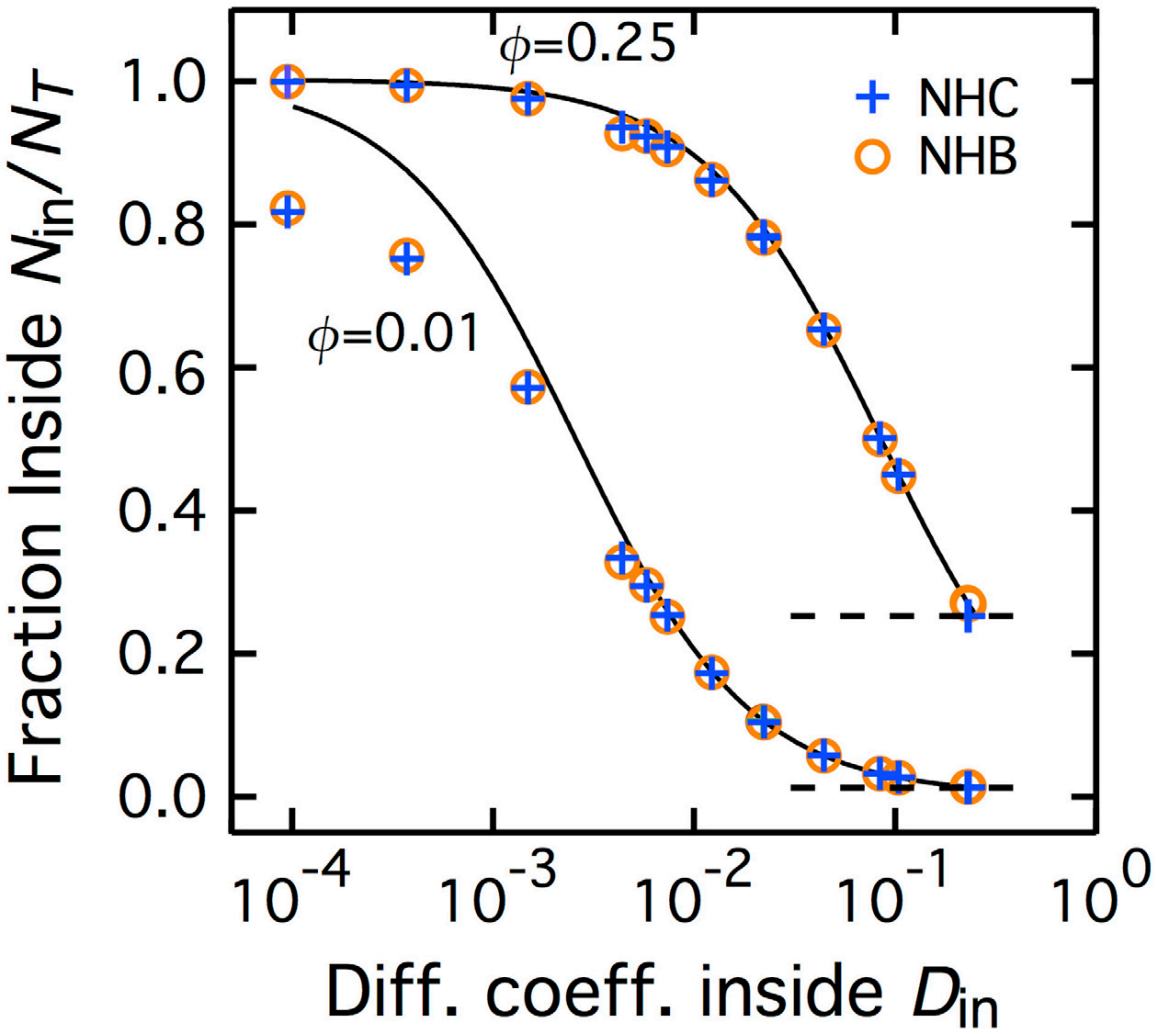
**Non-homogeneous CTRW and non-homogeneous Brownian yield identical accumulation inside the patch**. The fraction of molecules inside the central patch *N*_in_/*N*_T_ at equilibrium increases with the slowdown of diffusion inside the patch (where *N*_in_ and *N*_T_ are the number of molecules inside the central patch and the total number of molecules, respectively). NHC conditions are represented with blue plus signs whereas the NHB results are shown with brown empty circles. The full curves are the predictions from the theoretical expression for NHB, Equation (2) The dashed horizontal lines locate *N*_in_/*N*_T_ = 0.25 and *N*_in_/*N*_T_ = 0.01. The fraction area occupied by the central patch is ϕ = 0.25 (top curve) or 0.010 (bottom curve). In the NHC case, *D*_in_ is here the macroscopic diffusion coefficient of the transient CTRW inside the patch.

Figure 2 shows the average fraction of molecules present, at equilibrium, in patches of fraction area ϕ = 0.25 or 0.01. The case of non-homogeneous CTRW (NHC), where diffusion is Brownian outside the patch and a transient CTRW inside, is plotted with blue plus (+) signs. Each data point corresponds to a value of the CTRW parameter pair (γ,τ_*C*_). To facilitate visual presentation, we determined the macroscopic diffusion coefficient defined by each parameter pair (according to Equation 1) and plot the ratio of molecules inside the patch (at equilibrium) *N*_in_/*N*_T_ against the corresponding value of *D*_*M*_ inside the patch (*D*_in_).

For large values of *D*_in_, when diffusion is only weakly slowed down by the CTRW (τ*_C_* small and/or γ large), *N*_in_/*N*_T_ tends to ϕ (horizontal dashed lines), the fraction area of the patch. This is exactly the value expected when the densities of molecule inside and outside the patch are identical, *N*_in_/ϕ = *N*_out_/(1 − ϕ). Therefore, when the CTRW inside the patch has a short or weakly anomalous transient, the spatial distribution remains uniform and homogeneous. When the transient anomalous regime becomes more marked (*D*_in_ decreases), *N*_in_/*N*_T_ increases and becomes larger than ϕ. This reveals that with increasingly marked anomalous regimes (longer lasting or more anomalous), the equilibrium spatial distribution becomes non-homogeneous: molecules progressively accumulate inside the patch [*N*_in_/ϕ > *N*_out_/(1 − ϕ)]. For the smaller *D*_in_ values, the fraction of molecules inside the patch even tends to unity. In other words, when the cutoff time τ*_C_* tends to very large values, accumulation reaches extreme levels since roughly all the diffusing molecules are found inside the slowed down patch.

Therefore, when the duration of the anomalous transient regime is large and/or the anomalous exponent small, non-homogeneous CTRW (NHC) leads to accumulation at equilibrium. To investigate the origin of this accumulation, we compared the results obtained with NHC to those with non-homogeneous Brownian motion (NHB), in which the motion remains Brownian inside the patch, but with a smaller diffusion coefficient *D*_in_. In terms of mean-squared displacement 〈*R*^2^〉, NHB thus preserves the long-time behavior of NHC, but does not feature the initial anomalous transient. In a previous work (Soula et al., 2013), we showed that decreasing the ratio of diffusion coefficients *D*_in_/*D*_out_ in NHB, leads to accumulation at equilibrium inside the patch. We obtained a very good theoretical approximation of this accumulation with a simple expression:

(2) $$N_{\text{in}}/N_{\text{T}}=\frac{\phi }{\phi +\left(1-\phi \right)\frac{D_{\text{in}}}{D_{\text{out}}}}$$

Note in particular that in this expression, *N*_in_/*N*_T_ → ϕ when *D*_in_ → *D*_out_ (homogeneous distribution) while *N*_in_/*N*_T_ → 1 when *D*_in_ → 0 (total accumulation in the patch).

The brown open circles in Figure 2 show the accumulation obtained with NHB, for various values of *D*_in_ (and constant *D*_out_). The accumulation values obtained in simulations of NHB match almost exactly those obtained with NHC. To confirm this result, we also plot in Figure 2 the theoretical predictions of Equation (2) (full black lines). The agreement between this theoretical prediction and the simulation results, either for NHB or NHC, is almost everywhere very good, confirming match between NHB and NHC values. A discrepancy between the theoretical prediction and the simulation results (both for NHB or NHC) appears for very small patch fraction area (ϕ = 0.01) and strong slowdown (*D*_in_ < 10^−3^). This discrepancy might be due to the fact that, with such extreme slowdown in the patch, our maximal simulation time may be too short to reach equilibrium. In any case, this discrepancy does not invalidate the very good match for most of the values.

**Figure 3:**
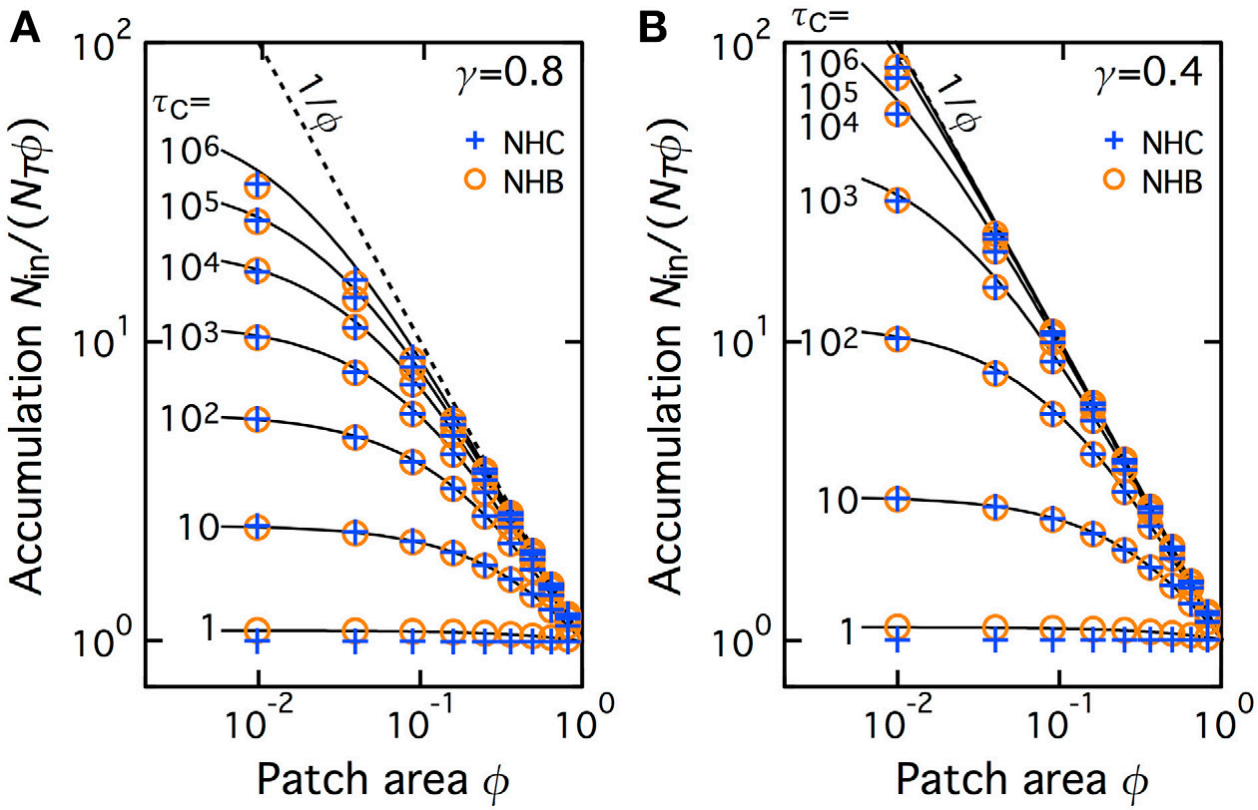
**Accumulation depends on patch area and the amplitude of the slowdown in the patch**. The extent of accumulation *N*_in_/(*N*_T_ϕ) ranges from 1.0 (no accumulation, *N*_in_/*N*_T_ = ϕ) for τ*_C_* = 1.0 (i.e., *D*_in_ = *D*_out_) to 1/ϕ (all the molecules are inside the patch), when τ*_C_* → ∞ (i.e., *D*_in_ → 0). The symbols are identical to those in Figure 2. The dashed line locates the curve *N*_in_/(*N*_T_ϕ) = 1/ϕ. The anomalous exponent γ = 0.8 **(A)** or 0.4 **(B)**.

We then extended this comparison to a larger set of values of the fraction area of the patch. To quantify the accumulation more directly, we plot on Figure 3 the values of *N*_in_/(*N*_T_ϕ) for various values of the CTRW parameters in the patch: cutoff τ*_C_* and anomalous exponent (γ = 0.8 in **A** and 0.4 in **B**). Using the same symbols as Figure 2 above, Figure 3 shows the results obtained with NHC and compare them to simulation and theoretical accumulation in a NHB with comparable *D*_in_. In absence of accumulation (i.e., with homogeneous spatial distribution of the molecules), one expects *N*_in_/(*N*_T_ϕ) ~ 1 whereas *N*_in_/(*N*_T_ϕ) should be close 1/ϕ (dashed line) when accumulation is close to complete in the patch. For both exponent values, the spatial distribution is found homogeneous (or close to homogeneous) for small cutoff times τ*_C_* and progressively converges to 1/ϕ when τ*_C_* → ∞. The limit of almost-complete accumulation (1/ϕ) is reached for smaller τ*_C_* when γ is small (thus diffusion heavy anomalous) than when γ is large. This confirms that NHC progressively leads to complete accumulation when the cutoff time increases. Here again the match with the simulated and theoretical values of NHB is very good.

**Figure 4:**
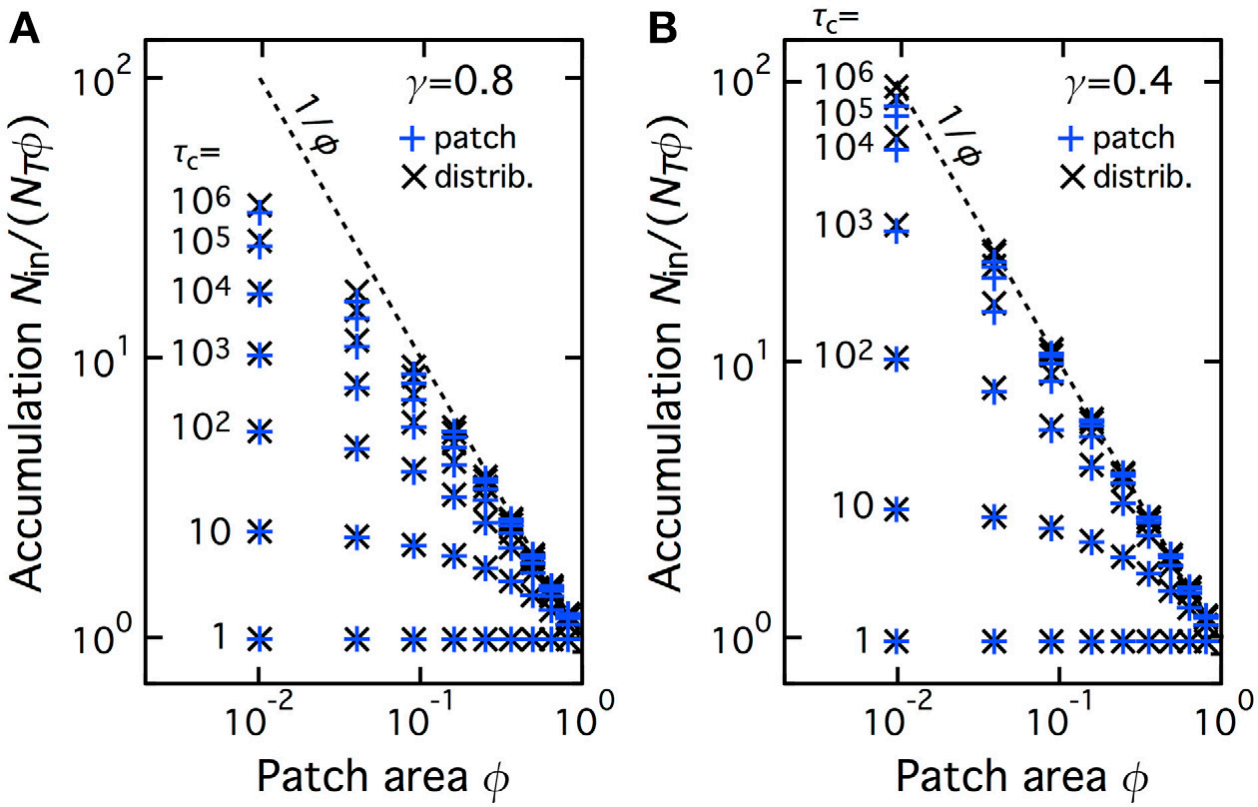
**The accumulation phenomenon with NHC is not specific of the central patch geometry**. Accumulation values are shown for simulations of NHC with a single central CTRW patch (i.e., the geometry used in Figure 3, blue plus signs, “patch” configuration) and when the patch is split into many smaller subparts, that are spread uniformly in the space domain (“distributed” configuration, black crosses). See the main text for details. All the other parameters and symbols are identical to Figure 3, in particular the anomalous exponent γ was 0.8 **(A)** or 0.4 **(B)**.

We next investigated whether this accumulation was specific to the geometry used in Figures 1–3. To this end we compared two geometries: (*i*) *Patch geometry*: CTRW takes place in a single patch of surface area ϕ*w*^2^ (where *w*^2^ is the total domain surface area), located in the center of the domain. This is the geometry used in Figures 1–3 above; (*ii*) *Distributed geometry*: The patch is split into *N_p_* squares of individual area *S* (we used unit area, *S* = 1); the total patch area is preserved (i.e., *N*_*p*_*S* = ϕ*w*^2^ ); the *N_p_* patches are distributed uniformly at random in the domain (without overlapping). Figure 4 plots the values of *N*_in_/(*N*_T_ϕ) for several CTRW parameter pairs (γ,τ*_C_*) obtained from Monte-Carlo simulations of the two geometries. Clearly, the amounts of accumulation observed for the two geometries, patch (blue “+” signs) or distributed (black “×” signs), are identical, for all the total patch areas and all the CTRW parameters we tested. Therefore, accumulation is also observed when the central patch is split into subpatches uniformly distributed in the domain. This indicates that the observed accumulation is a generic property of NHC that is not specific to the precise geometry configuration.

Those results therefore reveal that NHC leads to progressive accumulation of the molecules inside the central patch, whatever its area fraction. Since the amplitude of the accumulation due to NHC is almost identical to the amplitude of the accumulation due to NHB, one may conclude from those results that, at least regarding the spatial distribution of the molecules at equilibrium, the transient anomalous regime has no significant impact. Accumulation would therefore be entirely controlled by the “macroscopic” Brownian regime that is exhibited at long times by transient CTRW.

**Figure 5:**
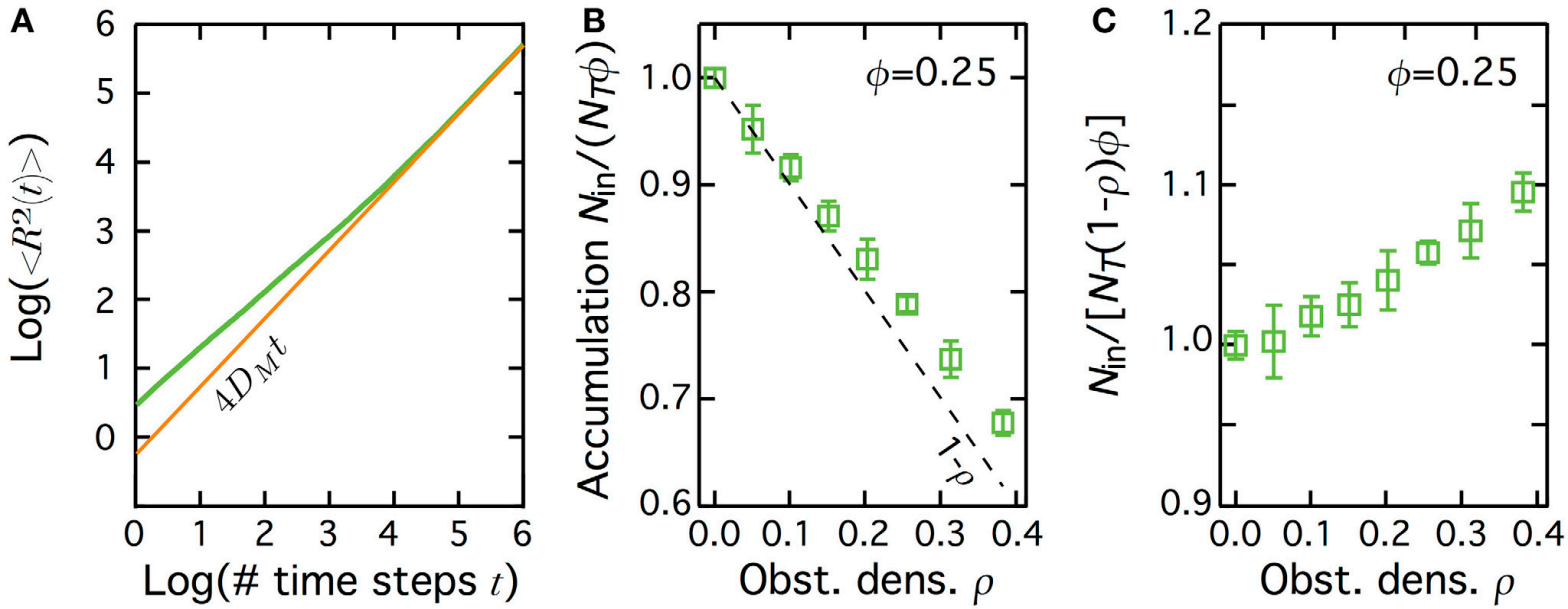
**Diffusion hindering by obstacles leads to depletion of the molecules in the patch at equilibrium**. **(A)** The mean square displacement 〈*R*^2^〉 in transient subdiffusion due to hinderance by randomly-located immobile obstacles (*green* curve) first scales as *t*^γ^ (with γ<1), before converging to a Brownian motion with *D_M_* a (macroscopic) diffusion coefficient, i.e., 〈*R*^2^〉 = 4*D_M_t* (*brown* curve). Obstacle density ρ = 0.35, patch area fraction ϕ = 1. **(B)** When the obstacles are restricted to a central patch, the number of molecules inside the patch at equilibrium decreases below 1.0, *N*_in_/(*N*_T_ϕ) ≤ 1.0 (depletion). The dashed line shows *N*_in_/(*N*_T_ϕ) = 1 − ρ. **(C)** When accumulation is computed using the effectively accessible area in the patch (1 − ρ)ϕ, and not the total patch area ϕ, one gets instead a weak accumulation in the patch. In **(B,C)**, bars indicate ± 1 s.d., and the fraction area of the patch ϕ = 0.25.

A convenient way to test this hypothesis is to compare the results obtained with transient subdiffusion due to obstacle hindering. In this case, diffusion is still Brownian-like inside the patch, except for the presence of randomly-distributed immobile obstacles that hinder diffusion (see Materials and Methods) (Saxton, 1994; Berry, 2002; Höfling and Franosch, 2013). In this case (*green* curve in Figure 5A), the mean-squared displacement 〈*R*^2^〉 is transiently anomalous with γ = 0.659 in 2D (in continuum space) (Bouchaud and Georges, 1990; Kammerer et al., 2008). Just like in transient CTRW, 〈*R*^2^〉 then converges to slowed-down Brownian motion with a “macroscopic” diffusion coefficient *D*_*M*_ (*brown* line in Figure 5A). When obstacle density ρ inside the patch increases, the crossover time from transient to Brownian regimes increases while *D*_*M*_ decays. Therefore, from the perspective of the mean-squared displacement, transient subdiffusion due to hindering by obstacles is very similar to transient CTRW. In particular, both exhibit a slowed-down Brownian behavior at long times.

Figure 5B shows the changes of *N*_in_/(*N*_T_ϕ) when the obstacle density is changed, for a central patch with fraction area ϕ = 0.25. Note that when immobile obstacles are added in the patch, some of the unobstructed sites of the patch find themselves isolated from the rest of the patch inside a cage made of obstacles. We found that in our simulations, those encaged sites were never present in a very significant amount (less than 9 % of the unobstructed patch sites, even at large obstacle densities). However, since they are not accessible for proteins located outside the cage, we included the fraction of such encaged isolated sites in the calculation of the obstacle density ρ. Without obstacles (ρ = 0), one recovers the expected spatially homogeneous distribution of the molecules at equilibrium [*N*_in_/(*N*_T_ϕ) = 1]. In a striking contrast with NHC or NHB, though, increasing the obstacle density in the patch (thus increasing τ*_C_* and decreasing *D*_in_) leads to a quick decay of the accumulated fraction below 1. This reveals that increasing the motion slowdown in the patch by obstacle hindering leads to a non-homogeneous equilibrium distribution of the molecules. But in opposition to the NHB or NHC case, this leads to a depletion of the molecule in the patch: the molecule density inside the patch becomes smaller than outside.

However, examination of Figure 5B shows that the values of *N*_in_/(*N*_T_ϕ) decrease slightly slower than the available area fraction inside the patch (1 − ρ, dashed line in the figure). This means that if one uses the accessible area in the patch [(1 − ρ)ϕ] to compute concentration (and not the total patch area ϕ), hindering by immobile obstacles in fact leads to a slight accumulation. To quantify this further, we show on Figure 5C the results obtained when we compute accumulation using the effectively accessible volume (1 − ρ)ϕ, i.e., we now compute accumulation as *N*_in_/(*N*_T_ (1 − ρ)ϕ). In agreement with the above comment, this figure shows a slight accumulation, especially for large obstacle densities. Note however that this accumulation effect is small as it never get larger than 10%.

Therefore, although they show very similar behaviors in terms of mean-square displacement, the impact on the equilibrium protein distribution in space of non-homogeneous transient subdiffusion due to obstacle hinderance or to CTRW are very different: while the latter leads to strong and robust accumulation in the patch, the former yields depletion or weak accumulation (depending on how concentrations are calculated).

## 4. Discussion

The lateral diffusion of proteins in membranes, and more generally, in the intracellular spaces is a complex process. First, many experimental reports evidence that their mobility does not agree with the classical Brownian motion but exhibit subdiffusion (Schwille et al., 1999; Smith et al., 1999; Fujiwara et al., 2002; Weigel et al., 2011; Höfling and Franosch, 2013). Moreover, the properties of their diffusion process can themselves vary from one location to another in the cell (or membrane) because of e.g., the non-homogeneous distribution of macromolecular crowding in space (Wachsmuth et al., 2000; Kühn et al., 2011; Parry et al., 2014) or the presence of nanodomains in the membranes that locally alter diffusion (Kenworthy et al., 2004; Goodwin et al., 2005; Fujita et al., 2007; Jacobson et al., 2007; Day and Kenworthy, 2009; Kusumi et al., 2011; Cambi and Lidke, 2012). Understanding protein mobility subject to such a complex process is still challenging. Here, we used Monte-Carlo simulations to study the spatial distribution (at equilibrium) of a protein that moves on a non-homogeneous two-dimensional domain with Brownian diffusion outside a central subregion (“patch”) and with transient CTRW inside (Figure 1C).

Our first finding is that in these conditions, proteins tend to accumulate inside the central patch (Figures 2, 3). When the anomalous regime becomes increasingly marked (i.e., the longer it lasts and the smaller its anomalous exponent), the density of proteins located at equilibrium inside the central patch is increasingly larger than the density of proteins outside. At the limit were the CTRW becomes permanent (i.e., when the cutoff time diverges), this accumulation is complete, i.e., close to all the mobile proteins are found inside the central patch. This result agrees with the one-dimensional case studied by Korabel and Barkai (2010, 2011); Fedotov and Falconer (2012), extending them to two-dimensional spaces and generalizing it to variable fraction areas for the CTRW patch and to transient CTRW.

Our second contribution is that in the case of non-homogeneous transient CTRW, this accumulation phenomenon is entirely driven by the Brownian regime that is reached after the transient anomalous regime. The anomalous exponent impacts the accumulation phenomenon because it sets the effective macroscopic diffusion coefficient in the Brownian regime, not because it causes the initial sublinear scaling of the mean square displacement with time. Accordingly, when we suppressed the initial transient anomalous regime keeping the same asymptotic Brownian one (i.e., simulating non-homogeneous Brownian motion), our simulations shows the same accumulation, quantitatively and qualitatively. However, we also show that this conclusion cannot be generalized to other mechanisms that cause transient subdiffusion. Indeed, the evolution with time of the mean square displacement when diffusion is hindered by randomly-located immobile obstacles exhibits the same changes with time as those of transient CTRW (compare Figure 5A with Figure 1A). Yet, when such a process is used to simulate non-homogenous transient subdiffusion, our simulations did not evidence strong local accumulation of the proteins. Therefore, two mechanisms for subdiffusion, CTRW and obstacle hindrance, can present exactly the same regimes for the changes of the mean-squared displacement (〈*R*^2^〉) with time, but lead to very different results regarding the protein distribution at equilibrium.

Our simulations do not account for excluded volume between diffusing proteins as several proteins can share the same lattice site at a given time. Including a hard limit on the maximal number of proteins that can share the same lattice site would lead to a significant decrease of the apparent diffusion coefficient (inside and outside the patch). This excluded volume interaction between proteins would arguably be more realistic but is also more demanding in terms of computation time. Moreover, we do not think it would change our main conclusions since excluded volume interaction between the proteins is not expected to modify their diffusion regime. However, it could of course have the trivial effect of limiting the amplitude of the reported accumulations inside the patch, since the bound on the number of proteins per lattice site implies a strict limitation of the number of proteins in the patch.

Taken together, our results provide a clear indication that the changes of 〈*R*^2^(*t*)〉 with time are not enough to explain the spatiotemporal dynamics of the proteins, even in the simple case, studied here, where the mobile protein to not interact via biochemical reactions or interactions. This realization has already started to emerge in the most recent experimental reports (see e.g., Parry et al., 2014). Other quantities that can be studied include quantifiers of the weak ergodicity breaking, specific to CTRWs (Burov et al., 2011; Weigel et al., 2011; Tabei et al., 2013) or quantifiers of how the distribution of the successive displacement deviates from a Gaussian distribution, that is expected for a Brownian motion (Parry et al., 2014). See e.g., Metzler et al. (2014) for a recent survey of those quantities. Moreover, CTRWs can give rise to spontaneous population splitting into mobile and immobile fractions (Schulz et al., 2013, 2014). This specificity of CTRWs could also be exploited to define more informative quantities.

Albeit the present work only concerns protein mobility, i.e., without coupling to a reaction, our results also shed new lights on the outcome of biochemical reactions when they occur among proteins with such non-homogeneous transient subdiffusion. In a recent article, we have studied the spatiotemporal dynamics of the ligand-binding equilibrium (L + R ⇌ C) (Soula et al., 2013) in a similar space-dependent setup. We compared the apparent affinity of the reaction when diffusion in the central patch is restricted by transient subdiffusion either due to obstacle hindrance or NHC or NHB. We found that, while CTRW systematically decreases the apparent affinity of the reaction i.e., makes it less likely to occur, both non-homogeneous Brownian motion and local hinderance by obstacles increase it. The improvement of the affinity with non-homogeneous Brownian motion seems expected due to accumulation inside the patch. However, this explanation fails in the case of transient CTRW. It also fails in the case of transient subdiffusion due to obstacles. Indeed albeit obstacle hindrance yields depletion of the proteins inside the patch, it still gives rise to a slight improvement in apparent affinity of the ligand binding equilibrium. Therefore, our main conclusions is that both equilibrium concentrations or the asymptotic behavior of the mean square displacement are not the key control for the dynamics of the ligand-binding equilibrium. It seems that even at equilibrium the structure of the anomalous transient, and/or other quantifiers of the mobility have a deep impact. Future works will be needed to understand those impacts.

## Author contributions

Hugues Berry and Hédi A. Soula conceived and designed the work; acquired, analyzed and interpreted the simulation data and wrote the paper.

### Conflict of interest statement

The authors declare that the research was conducted in the absence of any commercial or financial relationships that could be construed as a potential conflict of interest.
